# Modulating Human Mesenchymal Stem Cell Plasticity Using Micropatterning Technique

**DOI:** 10.1371/journal.pone.0113043

**Published:** 2014-11-17

**Authors:** Ajay Tijore, Feng Wen, Chee Ren Ivan Lam, Chor Yong Tay, Lay Poh Tan

**Affiliations:** 1 Division of Materials Technology, School of Materials Science and Engineering, Nanyang Technological University, Singapore, Singapore; 2 Department of Chemical and Biomolecular Engineering, National University of Singapore, Singapore, Singapore; Second University of Naples, Italy

## Abstract

In our previous work, we have reported that enforced elongation of human mesenchymal stem cells (hMSCs) through micropatterning promoted their myocardial lineage commitment. However, whether this approach is robust enough to retain the commitment when subsequently subjected to different conditions remains unsolved. This de-differentiation, if any, would have significant implication on the application of these myocardial-like hMSCs either as tissue engineered product or in stem cell therapy. Herein, we investigated the robustness of micropatterning induced differentiation by evaluating the retention of myocardial differentiation in patterned hMSCs when challenged with non-myocardial differentiation cues. Altogether, we designed four groups of experiments; 1) Patterned hMSCs cultured in normal growth medium serving as a positive control; 2) Patterned hMSCs cultured in normal growth medium for 14 days followed by osteogenic and adipogenic media for next 7 days (to study the robustness of the effect of micropatterning); 3) Patterned hMSCs (initially grown in normal growth medium for 14 days) trypsinized and recultured in different induction media for next 7 days (to study the robustness of the effect of micropatterning without any shape constrain) and 4) Patterned hMSCs cultured in osteogenic and adipogenic media for 14 days (to study the effects of biochemical cues versus biophysical cues). It was found that hMSCs that were primed to commit to myocardial lineage (Groups 2 and 3) were able to maintain myocardial lineage commitment despite subsequent culturing in osteogenic and adipogenic media. However, for hMSCs that were not primed (Group 4), the biochemical cues seem to dominate over the biophysical cue in modulating hMSCs differentiation. It demonstrates that cell shape modulation is not only capable of inducing stem cell differentiation but also ensuring the permanent lineage commitment.

## Introduction

Stem cells are undifferentiated self renewable progenitor cells that can develop into different specialized cell types [1], [2]. Previous studies have showcased the multipotency of adult stem cells along several lines of tissue lineages under the influence of well-defined biochemical concoctions [3]–[5]. From the developmental perspective, resident adult stem cells in tissues are observed to abide by lineage restriction, whereby their developmental fate is limited to cell types of their tissue of origin. For instance, hematopoietic stem cells (HSCs) continue the production of blood cells and skin stem cells give rise to keratinocytes. However stem cells are also known to differentiate into cell types different from their tissue of origin. Ferrari et al. reported that bone marrow-derived progenitors can generate muscle tissue in response to physiological stimuli [6]. Bone marrow stem cells were also found to differentiate into hepatic oval cells under certain physio-pathological condition [7]. This phenomenon termed as stem cell plasticity clearly showed that the differentiation fate of adult stem cells are not confined to developmental lineage restriction, but possibly modulated by their microenvironment. Cell plasticity is the ability of a cell to switch to another lineage that is not native to the tissue of origin. In the past, stem cell plasticity had not only created excitement but also posed doubts in the mind of researcher community. However, stem cell plasticity suggests an immense potential for therapeutic applications in regenerative medicine as adult stem cells from various tissue origins could be modulated through microenvironmental influences to develop into cells of vital tissues and organs of related or unrelated origin for replacement [8]–[12].

The fate of adult stem cells within its own niche is governed by a wide repertoire of regulatory factors such as cell-cell communication, cell-matrix adhesions, growth factors and other tissue-specific physiological parameters [13]–[16]. However evidences emerged in recent years suggest that cell shape could also be a potent biophysical regulator. Micropatterning is a widely used method to control stem cell fate by modulating the cell shape [17], [18]. Early studies by Folkman et al. used plastic culture dishes coated with different concentrations of poly(2-hydroxyethyl methacrylate) to control the cell shape and observed a strong correlation between cell shape and DNA synthesis [19]. McBeath et al. employed microcontact printing technique to modulate hMSCs commitment along the adipo- or osteogenic lineages, simply by controlling the cell shape on geometrically defined fibronectin micropatterns [20]. Recently, we have also shown that stem cell myogenic differentiation can be regulated by modulating cell shape through patterning on various substrates [17], [21].

Here we report that patterned hMSCs maintained their lineage commitment even after culturing in different tissue-specific induction media at a later stage. Patterned hMSCs conditioned in normal culture medium were subsequently cultured in osteogenic and adipogenic media respectively followed by further investigation of their lineage commitment. Strikingly, patterned hMSCs maintained their myocardial lineage commitment even when the cells were later exposed to osteogenic or adipogenic medium. In another condition, when patterned hMSCs were trypsinized and re-cultured in different induction media, the primed hMSCs were still found to positively express cardiomyogenic marker β-MHC (beta myosin heavy chain). This comprehensive study thus provided us with critical insights regarding the dominant effects of biophysical cue-cell shape here over the biochemical cues on hMSCs differentiation. Moreover, it was revealed that patterned hMSCs are no longer plastic i.e. cells lost their “stemness” and committed to myocardial lineage.

## Materials and Methods

### Micropatterning of hMSCs

Cell micropatterning was performed as previously described [22]. Briefly, polydimethylsiloxane (PDMS) elastomeric patterned stamps bearing 20 µm elevated lanes and 40 µm spacing were fabricated and utilized to ink the human plasma derived fibronectin (BD Biosciences) onto glutaraldehyde (Sigma) treated cover glasses [18]. Cover glasses (22 mm diameter) were treated with piranha solution followed by immersion in 3% (3-aminopropyl) triethoxysilane (APTES) (Sigma) for 1 h and subsequent rinsing with ultrapure water for several times. Treated cover glasses were soaked in 2.5% glutaraldehyde solution for an hour and washed with ultrapure water. Fibronectin dissolved in phosphate buffer saline (PBS) solution (50 µg/mL) was added on the patterned surface of PDMS stamp and incubated for 1h followed by blow drying with pressurized nitrogen gas. Fibronectin coated PDMS stamps were apposed to modified cover glasses for an hour. Freshly prepared 2% bovine serum albumin (BSA) (Invitrogen) was used for blocking the nonspecific cell adhesion. Cover glasses were UV sterilized for 15 min followed by cell seeding with a density of 1–2×10^3^ cells per cm^−2^. Once sufficient cells were attached to the fibronectin strip pattern, cell culture medium containing unattached cells was replaced. Cell culture medium was replaced every 2–3 days.

### Cell Culture

hMSCs (Lonza, (Cambrex)) obtained from human bone marrow were used for experimental work. Low glucose Dulbecco’s Modified Eagle’s Medium (DMEM) containing L-glutamine (Sigma Aldrich) supplemented with 10% FBS (PAA) and 1% antibiotic/antimycotic solution (PAA) was used as the normal growth medium. Adipogenic medium was composed with normal growth medium supplemented with 1 µM dexamethasone, 200 µM indomethacin, 10 µg/mL insulin and 0.5 mM methylisobutylxanthine. Osteogenic medium was composed with normal growth medium consists comtaining 50 µM ascorbic 2-phosphate, 10 mM glycerophosphate and 100 nM dexamethasone. Cells were grown in an incubator at 37°C with humidified atmosphere of 5% CO_2_. For cell detachment, 0.25% trypsin-EDTA (Invitrogen) solution was used. Early passage (P5) hMSCs were utilized in all experimental studies. Four groups of experiments were performed; 1) Patterned hMSCs cultured in normal growth medium for 14 days; 2) Patterned hMSCs cultured in normal growth medium for 14 days followed by osteogenic and adipogenic media for the next 7 days; 3) Patterned hMSCs (initially grown in normal growth medium for 14 days) re-cultured in different growth media for 7 days after trypsinization and 4) Patterned hMSCs cultured in osteogenic and adipogenic media for 14 days.

### Immunofluorescence staining

All samples were fixed in 4% paraformaldehyde solution for 10 min and permeabilized with 0.1% Triton X-100 for 5 min prior to 1 h blocking with 5% BSA in PBS. Cell samples were incubated overnight with primary antibodies, mouse monoclonal anti-human cardiac myosin heavy chain (1∶400, Abcam) at 4°C followed by washing with PBS. Samples were then labeled with Alexa Fluor 488 goat anti mouse IgG (1∶400, Molecular Probes). 4′-6-diamidino-2-phenylindole (DAPI) (1∶400, Chemicon) was added to counterstain the cell nuclei. For osteocalcin staining, cells were stained with primary antibodies, rabbit polyclonal anti-osteocalcin (1∶200, Santa Cruz Biotechnology) followed by staining with Alexa Fluor 568 goat anti rabbit IgG (1∶100, Invitrogen). Images were captured with an Eclipse 80i upright microscope (Nikon) using 20x and 10x objective lenses and ImageJ 1.44f software was used for image analysis. For oil Red O dye staining, cell samples were fixed with 4% paraformaldehyde for 10 min and stained with oil red O dye solution for 15 min. Cell samples were subjected to scrutiny under an inverted microscope (Nikon eclipse TS100) using 20x and 10x objective lenses to observe oil globules formation.

### Statistical analysis

A statistical study was performed on a total of 300 cells each from patterned and unpatterned groups and values are mentioned as mean ± standard deviation (SD). One-way ANOVA method was used to determine the significant difference (*p*<0.05) within experimental groups.

## Results and Discussion

**Figure 1:**
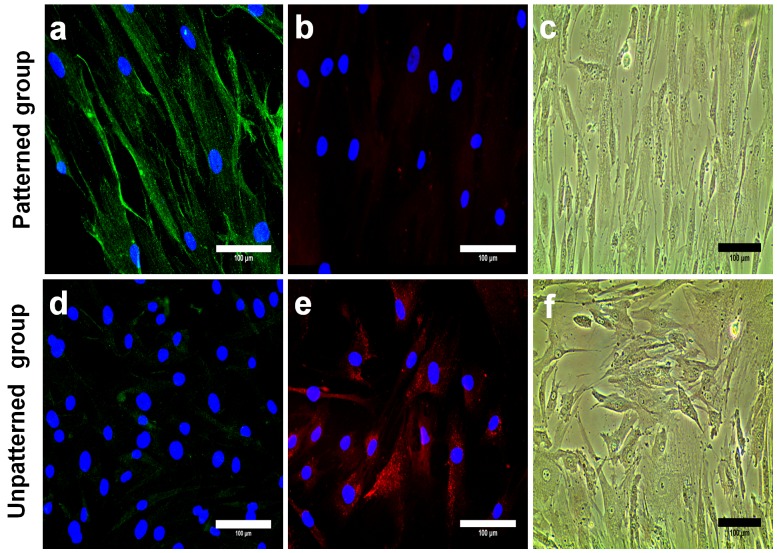
Immunostaining of β-MHC and osteocalcin along with oil droplets detection after 2 weeks of hMSCs culture in normal growth medium. Distinct up-regulation of β-MHC, a cardiac marker (green) was observed in the patterned cells (**a**) but not for the unpatterned cells (**d**). No signs of osteocalcin expression (**b**) and oil droplet formation (**c**) were observed in cells from patterned group. Unpatterned cells expressed osteocalcin abundantly (red) (**e**), while no oil droplets formation was observed in unpatterned cells (**f**). The scale bar is 100 µm.

Efficiency of cell micropatterning procedure was validated by microscopic examination of cell morphology. Cells grown on the micropatterned surface were highly elongated in contrast to cells cultured on unpatterned surface where cells were observed to take on a wide spectrum of shapes (Figure S1 in File S1). In the first group of experiments, we ascertained lineage commitment of both patterned and unpatterned cells by performing the immunofluorescence staining after 14 days of cell culture in normal growth medium (Figure 1). After 14 days of culture in normal growth medium, patterned cells showed prominent up-regulation of β-MHC (isoform of MHC), a mature marker of cardiomyogenesis (Figure 1a). It is a well known fact that β-MHC resides in human ventricular myocardium abundantly which strongly supported our claim that patterned cells are committed to the myocardial lineage. In addition, we used another mature cardiomyogenic marker, cardiac troponin T (cTnT) to reconfirm the myocardial lineage commitment of patterned cells (Figure S2 in File S1). Unpatterned cells contrastingly showed very weak β-MHC expression (Figure 1d). The fact of up-regulation of myocardial marker β-MHC in patterned cells is consistent with our past findings, where we performed the real-time polymerase chain reaction (PCR) to check the gene expression profile [19]. We observed a higher expression of several cardiomyogenic genes (MyoD, MyF5, GATA4, NKX2-5, MHC and cTnI) and down-regulation of osteogenic genes (ALPL, RUNX2, ON) in patterned cells grown on poly(lactic-co-glycolic acid) (PLGA) films with similar fibronectin strip pattern. Both patterned and unpatterned cell groups were also assessed for osteogenic and adipogenic lineage commitment. Osteocalcin is a non-collagenous protein abundantly secreted by osteoblasts for deposition in the mineralized adult bone. Hence, osteocalcin becomes a reliable positive indicator for osteogenic lineage commitment. Oil red O, a fat soluble dye is widely used for neutral triglycerides and lipids staining and to observe the formation of oil globules in fat cells. Also, RUNX2, an early marker osteogenesis and PPARγ, an adipogenic marker were used to further validate osteogenic and adipogenic lineage commitment (Figure S2 in File S1). Negative staining of osteocalcin (Figure 1b) and oil globules formation (figure 1c) was observed for the patterned cells, which indicated the absence of osteogenic and adipogenic lineage commitment in the patterned cells. In contrast, unpatterned cells showed the positive staining of osteocalcin (Figure 1e), whereas no sign of oil globules formation was noted in unpatterned cells (figure 1f). This result is not surprising as it has been observed that substrate stiffness can drive cell commitment toward the particular tissue lineage [23]. Hence cells grown on hard substrates like cover glasses tend to commit toward the osteogenic lineage and not the adipogenic lineage.

**Figure 2:**
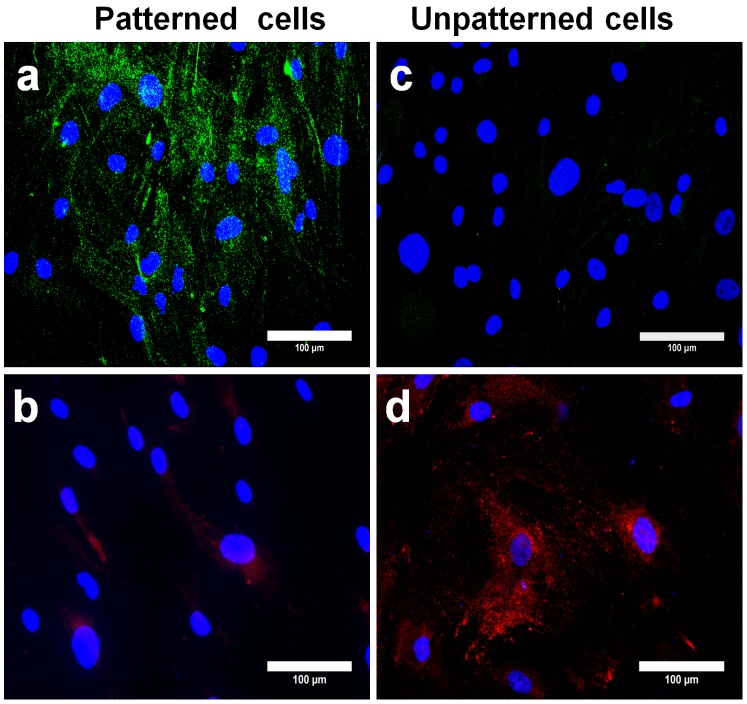
Immunostaining of β-MHC and osteocalcin after 21 days of hMSCs culture (14 days in normal growth medium +7 days in osteogenic medium). Patterned cells showed positive expression of β-MHC (**a**), whereas unpatterned cells stained negatively for β-MHC expression (**c**). Patterned cells showed negligible signs of osteocalcin expression (**b**) but significant increase in the level of osteocalcin expression was visualized in unpatterned cells (**d**). The scale bar is 100 µm.

**Figure 3:**
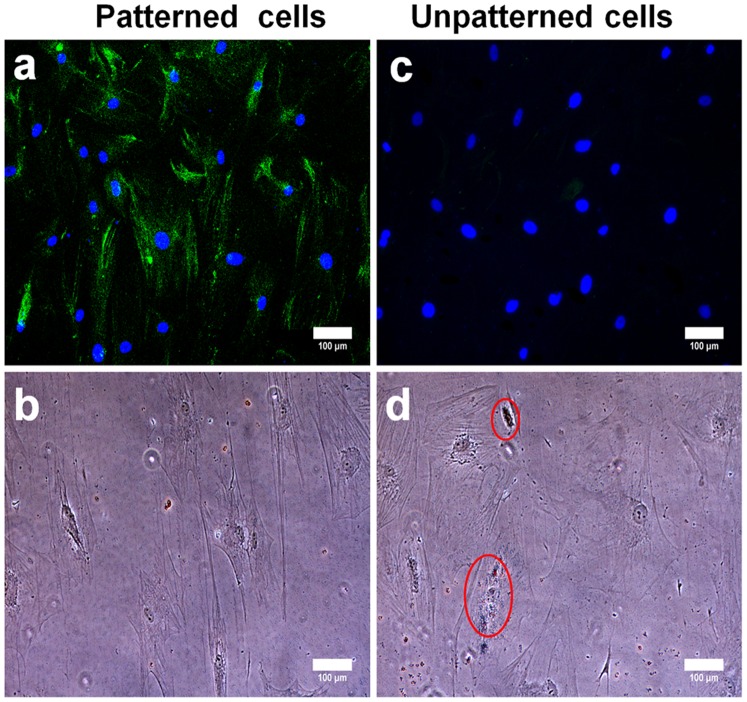
Immuno-detection of β-MHC and the detection of oil droplets after 21 days of hMSCs culture (14 days in normal growth medium +7 days in adipogenic medium). β-MHC was found to be up-regulated in patterned cells (**a**). In contrast, no indication of β-MHC expression was observed in unpatterned cells (**c**). Optical images of cells from both groups proved that elongated patterned cells failed to produce oil droplets (**b**). On the other hand, tiny oil globules were detected in unpatterned cells (**d**). The scale bar is 100 µm.

**Figure 4:**
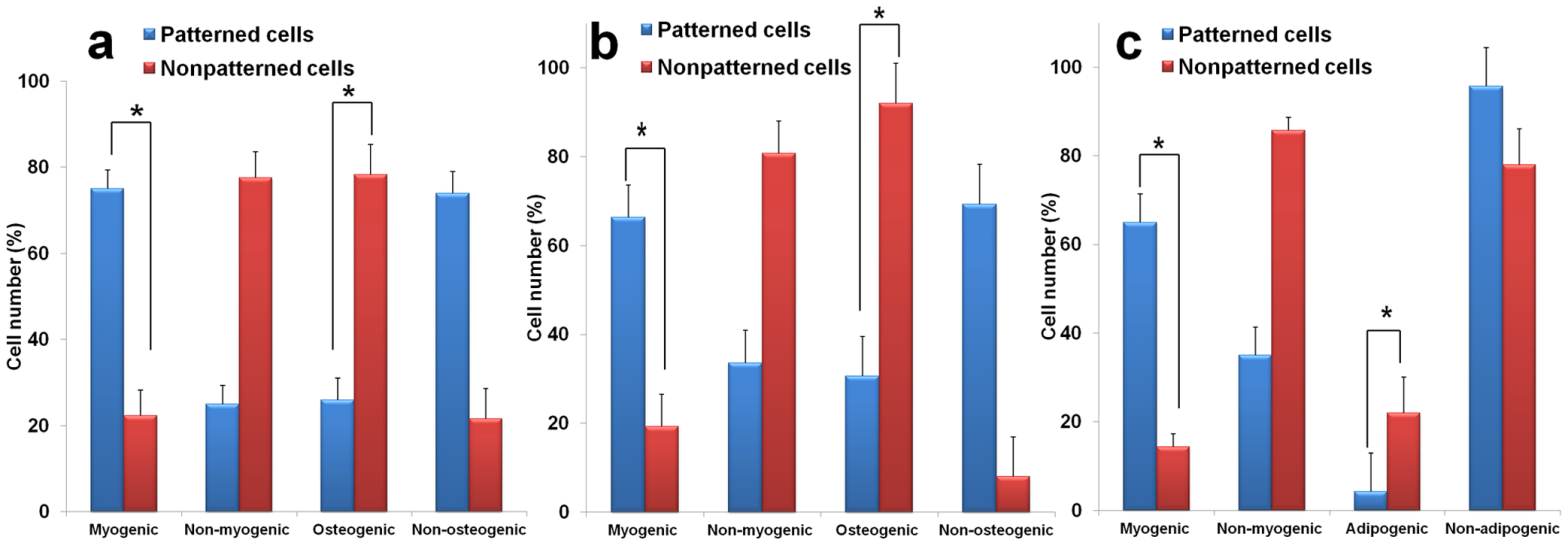
Statistical data illustrating the number of hMSCs committed to different tissue-specific lineages after total 21 days of cell culture. Cells were grown for 21 days in normal growth medium (**a**), 14 days in normal growth medium +7 days in osteogenic medium (**b**) and 14 days in normal growth medium +7 days in adipogenic medium (**c**). Bar represents the means ± standard deviation (SD) and asterisks indicate *p*<0.05 between the patterned and unpatterned groups.

In the second group of experiments, patterned and unpatterned cell groups were cultured initially in normal growth medium over a period of 14 days followed by replacement with osteogenic medium for further 7 days of culture. Afterwards, cells were immunolabeled for β-MHC and osteocalcin to determine the lineage commitment of cells. Similarly, cells were checked for CTnT and RUNX2 expression to reaffirm the lineage commitment (Figure S3 in File S1). Immunofluorescent images (Figure 2a & 2b) illustrated that about 66% of patterned cells retained myocardial lineage commitment by showing positive β-MHC staining, while negligible osteocalcin expression was detected. In contrast, 92% of unpatterned cells showed significant osteocalcin expression and no β-MHC expression indicating osteogenic lineage commitment (Figure 2c & 2d). Similarly, patterned and unpatterned cells were cultured in normal growth medium for 14 days and subsequently in adipogenic medium for consecutive 7 days. Further investigation of stem cell lineage commitment revealed that 65% of patterned cells maintained their myocardial lineage commitment with significant β-MHC expression and absence of oil globules formation (Figure 3a & 3b). Staining results of unpatterned cells were not in accord with patterned cells where it displayed negative β-MHC expression and also signaled oil globules formation, suggesting the adipogenic induction (Figure 3c & 3d). Moreover, immunostaining of cTnT and PPARγ was performed to check the cell lineage commitment and results observed were consistent with the findings mentioned above (Figure S4 in File S1). The results of percentage of cells committed to different tissue lineages (observed in second group of experiments) were compared to the control where patterned cells were cultured in normal growth medium for consecutive 21 days and a similar trend was observed in all three sets of experiments (Figure 4).

Taken together, the aforementioned studies demonstrated a general trend that hMSCs are extremely sensitive toward biophysical and biochemical cues in surrounding microenvironment and importantly it is reflected during cell differentiation. When cells were constrained to 20 µm wide fibronectin patterns in the presence of normal growth medium, up-regulation of cardiomyogenic genes expression was observed, while genes expression linked to other lineage commitment were suppressed distinctly [18]. Even after replacement of normal growth medium with other tissue lineage induction media (osteogenic/adipogenic), patterned cells maintained myocardial lineage commitment. This suggests that patterned cells have committed to myocardial lineage and are no longer plastic. Previous reports also strongly imply that modulation of cell shape and focal adhesions can be used as driving force to coax the myocardial differentiation [24]–[28].

**Figure 5:**
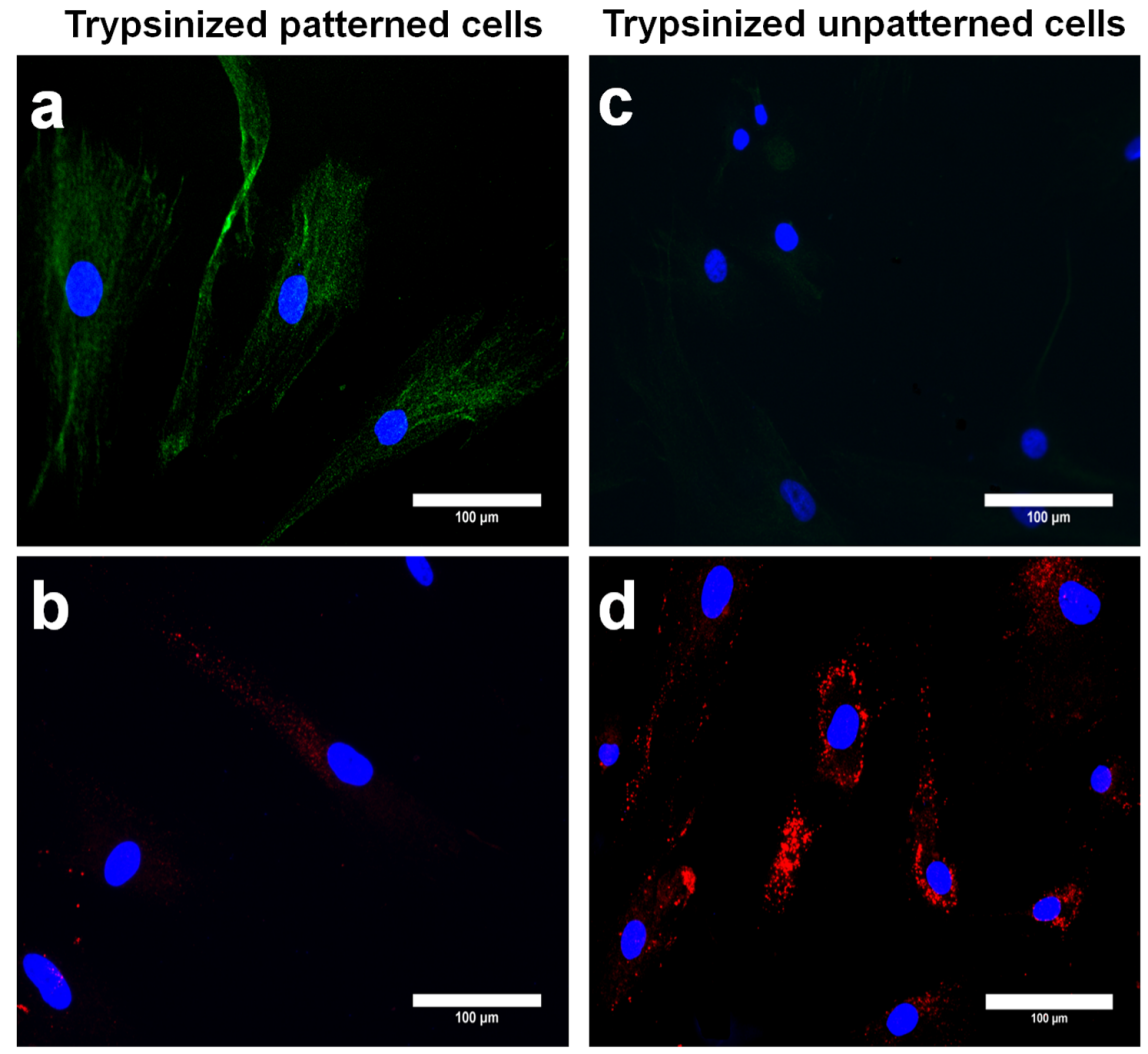
Verification of tissue-lineage commitment of trypsinized and re-cultured hMSCs (re-cultured in normal growth medium). Trypsinized hMSCs were re-cultured in normal growth medium for 7 days and subjected to immunostaining procedure. Trypsinized cells maintained their myocardial lineage commitment and it was confirmed by β-MHC positive immuno-labeling (**a**) and no osteocalcin expression (**b**). Contrary to that, trypsinized unpatterned cells showed down regulation of β-MHC (**c**) and predominant expression of osteocalcin (**d**).

**Figure 6:**
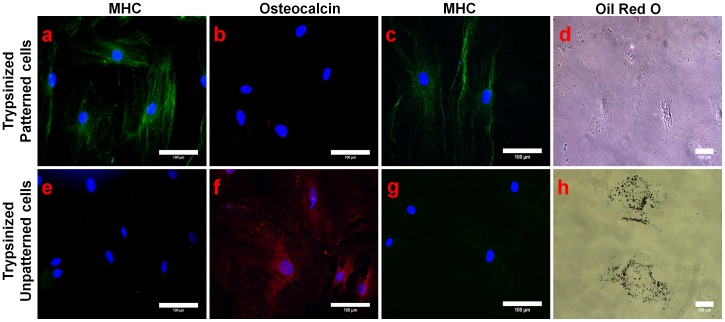
Investigation of tissue-lineage specificity of trypsinized and re-cultured hMSCs (re-cultured in osteo- and adipogenic induction medium). Trypsinized hMSCs (group 3) were re-cultured in osteogenic (**a, b, e, f**) and adipogenic medium (**c, d, g, h**) for 7 days and lineage commitment was validated by detecting the tissue-lineage specific markers; β-MHC, osteocalcin along with oil red O dye. Immunostaining results proved that trypsinized and re-cultured patterned cells exhibited cardiac MHC expression (**a, c**) in osteo- and adipogenic medium respectively, whereas these re-cultured patterned cells failed to express osteogenic marker (**b**) or to produce oil globules (**d**). Trypsinized and re-cultured unpatterned cells exhibited no traces of cardiac marker expression (**e, g**) but showed osteocalcin expression (**f**) and tiny oil globules formation (**h**). The scale bar is 100 µm.

In the third group of experiments, we would like to answer the question of whether the effect of micropatterning on the lineage commitment is robust enough to be retained even after the cells were trypsinized and re-plated onto unpatterned surfaces. To do that, committed hMSCs grown in normal growth medium (for 14 days) were trypsinized and reseeded onto conventional fibronectin coated cover glasses for further 7 days in the presence of normal growth medium, osteogenic medium and adipogenic medium respectively. Morphology of trypsinized cells (especially trypsinized patterned cells) was taken into consideration to see whether there are any morphological changes after they were exposed to various culture media and are shown in Figure S5 in File S1. Trypsinized patterned cells exhibited an elongated morphology in all three culture media suggesting that the cells were able to ‘remember’ previously assumed shape by maintaining their muscle-like phenotype. In contrast, unpatterned cells acquired various random shapes. It clearly indicated that trypsinized patterned cells tend to retain elongated cell morphology which might be crucial for the maintenance of myocardial lineage commitment. Our observations were supported by a recent study showing that hMSCs retained mechanical memory of its former growth environment. In addition, it was shown that the transcriptional coactivators YAP and TAZ played a deterministic role in storing this information from the mechanical environment which can critically shape the stem cell’s future fate [29]. Thus, trypsinized and recultured cell samples from both patterned and unpatterned groups were assessed for β-MHC, osteocalcin expression and oil globules formation respectively to determine the lineage commitment. Remarkably, immunostaining data illustrated that trypsinized and recultured patterned cells grown in normal growth medium sustained prominent cardiac MHC expression and very weak osteocalcin expression, emphasizing the maintenance of patterned cell’s myocardial lineage commitment (Figure 5a & 5b). Yet again, unpatterned cells did not show any significant expression of cardiac MHC, but visible osteocalcin expression was observed, indicative of osteogenesis (Figure 5c & 5d). Similarly, trypsinized and recultured patterned cells grown respectively in osteogenic and adipogenic media for 7 days stained positively for cardiac MHC (Figure 6a & 6c), while no traces of osteocalcin expression and oil globules were observed in those recultured patterned cells (Figure 6b & 6d). Myocardial lineage commitment of trypsinized and recultured patterned cells grown in different media was reconfirmed with cTnT immunostaining results (Figure S6 in File S1). Trypsinized and recultured unpatterned cells grown in osteogenic and adipogenic medium respectively did not express cardiac MHC (Figure 6e & 6 g). However, these recultured unpatterned cells showed distinct osteocalcin expression and oil globule formation in osteogenic and adipogenic medium respectively, indicating that osteogenic and adipogenic lineage commitment is dependent on induction medium used (Figure 6f & 6 h).

Altogether, our results suggest that myocardial lineage commitment of patterned cells was unaffected by cell trypsinization process. These patterned cells are thus said to be permanently committed along the myocardial lineage and have lost the signature of stemness i.e. plasticity. As we have seen, retention of elongated cell shape of the trypsinized and recultured patterned cells obviously showed a strong correlation between cell elongation and myocardial lineage commitment of patterned cells. Since cytoskeleton organization is actively engaged in cell signal transduction and mechanically couples the extracellular matrix to nucleus [30], [31], elongated cell morphology results in cytoskeleton reorganization and generate optimal cellular tension to induce the myocardial lineage commitment [24], [25].

**Figure 7:**
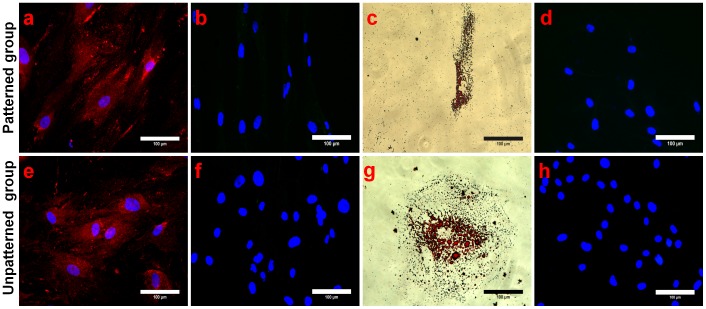
Validation of tissue-lineage commitment of hMSCs. Tissue-lineage specific markers; osteocalcin and β-MHC along with oil red O dye were used to check hMSCs commitment grown in osteogenic (**a, b, e, f**) and adipogenic medium (**c, d, g, h**) for 14 days. Immunostaining results revealed that patterned cells displayed osteocalcin expression (**a**) and oil globules formation (**c**) cultured in osteo- and adipogenic medium respectively, while patterned cells grown in osteo- and adipogenic medium failed to express cardiac marker (**b, d**). Unpatterned cells followed similar trend by showing osteocalcin expression (**e**), distinct oil globules formation (**g**) and no indication of β-MHC expression (**f, h**). The scale bar is 100 µm.

In the fourth group of experiments, we explored the effect of biophysical cues in the presence of different biochemical cues. In this experiment, stem cells from both patterned and unpatterned groups were cultured all throughout in osteogenic and adipogenic media respectively for 14 days. By day 14, patterned cells cultured in osteogenic medium were immunostained with cardiac and osteogenic markers to determine the lineage commitment. Interestingly, patterned cells not only expressed osteocalcin significantly but also a drastic decrease in β-MHC expression was observed (Figure 7a & 7b). Unpatterned cells also followed a similar trend with abundant osteocalcin expression and no positive cardiac marker staining (Figure 7e & 7f). This indicates the dominance of biochemical cues over the biophysical cues in directing stem cell differentiation. Similarly, in the context of adipogenic medium, patterned cells exhibited oil globules formation as well as no expression of cardiac marker (Figure 7c & 7d). This was also observed for the unpatterned cells (Figure 7 g & 7 h). Immunostaining results of RUNX2 and PPARγ markers expression also supported above mentioned results (Figure S7 in File S1). Substantial morphological differences were observed in these cells as displayed in Figure S8 in File S1. Patterned cells grown on fibronectin pattern substrates in normal growth medium (group 1) maintained their typical elongated morphology and run parallel to each other in close proximity. In comparison, cells cultured on the fibronectin pattern substrates in both osteogenic and adipogenic media (group 4) showed random cell morphology and negligible conformation to the lane patterns. In this study, the results indicated that the biochemical cues could be more dominant in modulating the hMSCs differentiation. We believe that this could be due to the inability of cell to develop the necessary focal adhesions and subsequently conform to the fibronectin patterns fast enough to counter the biochemical effect. Consequently, the biochemical cues dominated in directing the subsequent signaling into respective lineages.

The clinical applications of hMSCs in tissue engineering and repairing have been extensively explored before. It has been shown that dental pulp stem cells (DPSCs) loaded on the collagen scaffold can be used to repair the human mandible bone defects successfully [9]. Moreover, after few years of the grafting intervention in a same patient, DPSCs were able to regenerate the compact bone at the graft site [11]. These above mentioned clinical studies highlighted the potential of DPSCs in the bone tissue reconstruction engineering [12]. Also, researchers demonstrated that human adipose stem cells (positive for pericytic markers) cultured on cross-linked hyaluronic acid scaffolds showed a successful regeneration of human skeletal muscle in vivo [10]. Broadly speaking, these clinical studies along with our cardiomyogenic commitment results emphasize the importance of hMSCs derived from different origins in the field of tissue engineering and regenerative medicines.

## Conclusion

In conclusion, we demonstrated that patterned hMSCs permanently retained their myocardial lineage commitment even after post treatment with conflicting biochemical cues (i.e. adipogenic and osteogenic induction media) and trypsinization treatment. This study illustrates the importance of cell shape modulation in inducing stem cell differentiation and permanent lineage commitment. We believe that these findings are crucial for understanding the stem cell biology fundamentals underlying cellular cardiomyoplasty. Successful conditioning of hMSCs to cardiomyocytes compatible for cellular cardiomyoplasty using proper biophysical cues would be a breakthrough for prospective heart disease treatment. At this moment, we are still investigating the level of cardiomyogenic maturity of patterned hMSCs, but certainly, there is an enormous scope of improvement to further push cardiomyogenic maturity of these cells if proper biophysical cues, as conveyed here by cell shape distortion or other parameters like focal adhesion modulation can be regulated optimally. In addition, this study reinforces the advantage of using micropatterning technique in modulating stem cell differentiation and could be incorporated as part of the scaffold design for cardiac tissue engineering.

## Supporting Information

File S1 **Supporting files. Figure S1**, hMSCs were cultured on the cover glasses coated with 20 µm wide fibronectin printed pattern and evenly distributed fibronectin separately. **Figure S2**, Immunofluorescence staining of cardiac troponin T (cTnT), RUNX2 and PPARγ after 2 weeks of cell culture in normal growth medium. **Figure S3**, Immunofluorescence staining of cardiac troponin T and RUNX2 after 21 days of hMSCs culture (14 days in normal growth medium +7 days in osteogenic medium). **Figure S4**, Immunodetection of cardiac troponin T and PPARγ after 21 days of hMSCs culture (14 days in normal growth medium +7 days in adipogenic medium). **Figure S5**, Morphology of trypsinized and re-cultured hMSCs (group 3) from patterned and unpatterned groups were displayed. **Figure S6**, Validation of myocardial lineage commitment of trypsinized and re-cultured hMSCs using cardiac troponin T marker (re-cultured in normal growth medium, osteogenic and adipogenic induction medium respectively). **Figure S7**, Investigation of tissue-lineage commitment of hMSCs grown in osteogenic and adipogenic induction media for 2 weeks. **Figure S8**, Observing the morphological differences in hMSCs from both patterned and unpatterned groups.
(DOC)
