# Supplementary material for: Modulating Human Mesenchymal Stem Cell Plasticity Using Micropatterning Technique
Source: PLoS One. 2014 Nov 17;9(11):e113043. doi: 10.1371/journal.pone.0113043 (PMC4234627; doi:10.1371/journal.pone.0113043)
Supplement: File S1 — Supporting files. Figure S1, hMSCs were cultured on the cover glasses coated with 20 µm wide fibronectin printed pattern and evenly distributed fibronectin separately. Figure S2, Immunofluorescence staining of cardiac troponin T (cTnT), RUNX2 and PPARγ after 2 weeks of cell culture in normal growth medium. Figure S3, Immunofluorescence staining of cardiac troponin T and RUNX2 after 21 days of hMSCs culture (14 days in normal growth medium +7 days in osteogenic medium). Figure S4, Immunodetection of cardiac troponin T and PPARγ after 21 days of hMSCs culture (14 days in normal growth medium +7 days in adipogenic medium). Figure S5, Morphology of trypsinized and re-cultured hMSCs (group 3) from patterned and unpatterned groups were displayed. Figure S6, Validation of myocardial lineage commitment of trypsinized and re-cultured hMSCs using cardiac troponin T marker (re-cultured in normal growth medium, osteogenic and adipogenic induction medium respectively). Figure S7, Investigation of tissue-lineage commitment of hMSCs grown in osteogenic and adipogenic induction media for 2 weeks. Figure S8, Observing the morphological differences in hMSCs from both patterned and unpatterned groups. (DOC) [file pone.0113043.s001.doc]

**Supplementary Information**

**­Modulating Human Mesenchymal Stem Cell Plasticity using Micropatterning Technique**

Ajay Tijore^1^, Feng Wen^1^, Chee Ren Ivan Lam^1^, Chor Yong Tay^2^, Lay Poh Tan^1^ *

**^1^** Division of Materials Technology, School of Materials Science and Engineering, Nanyang Technological University, 50 Nanyang Avenue, 639798, Singapore

**^2^** Department of Chemical and Biomolecular Engineering, National University of Singapore, 4 Engineering Drive 4, 117585, Singapore

[*] E-mail: lptan@ntu.edu.sg


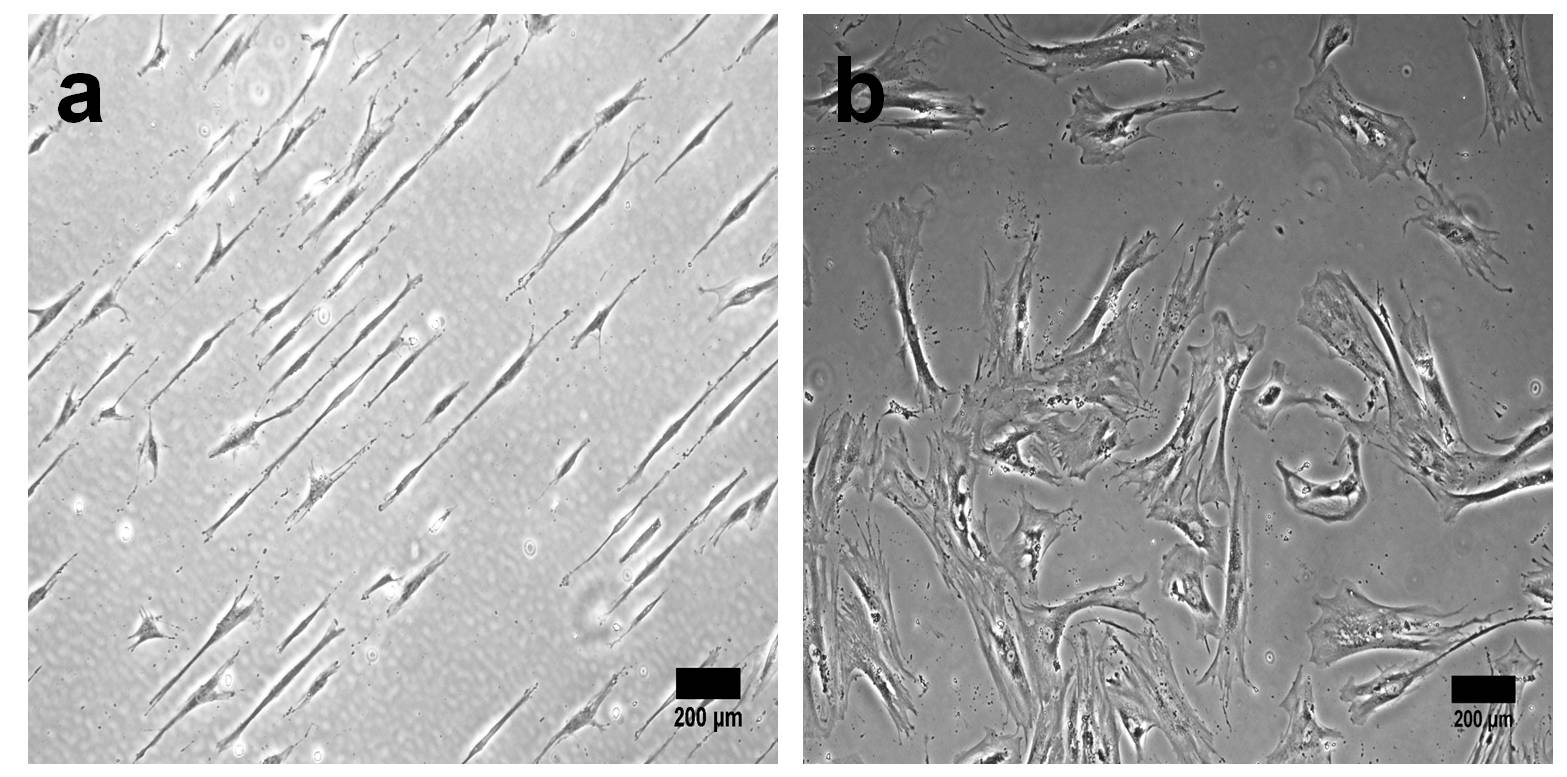


**Figure S1. hMSCs were cultured on the cover glasses coated with 20 µm wide fibronectin printed pattern and evenly distributed fibronectin separately.** Images of cells with elongated morphology on fibronectin strip pattern **(a)** and randomly grown cells on evenly coated fibronectin surface **(b)** were captured on day 3. The scale bar is 200 µm.

**Immunofluorescence staining using additional adipogenic, cardiomyogenic and osteogenic markers**


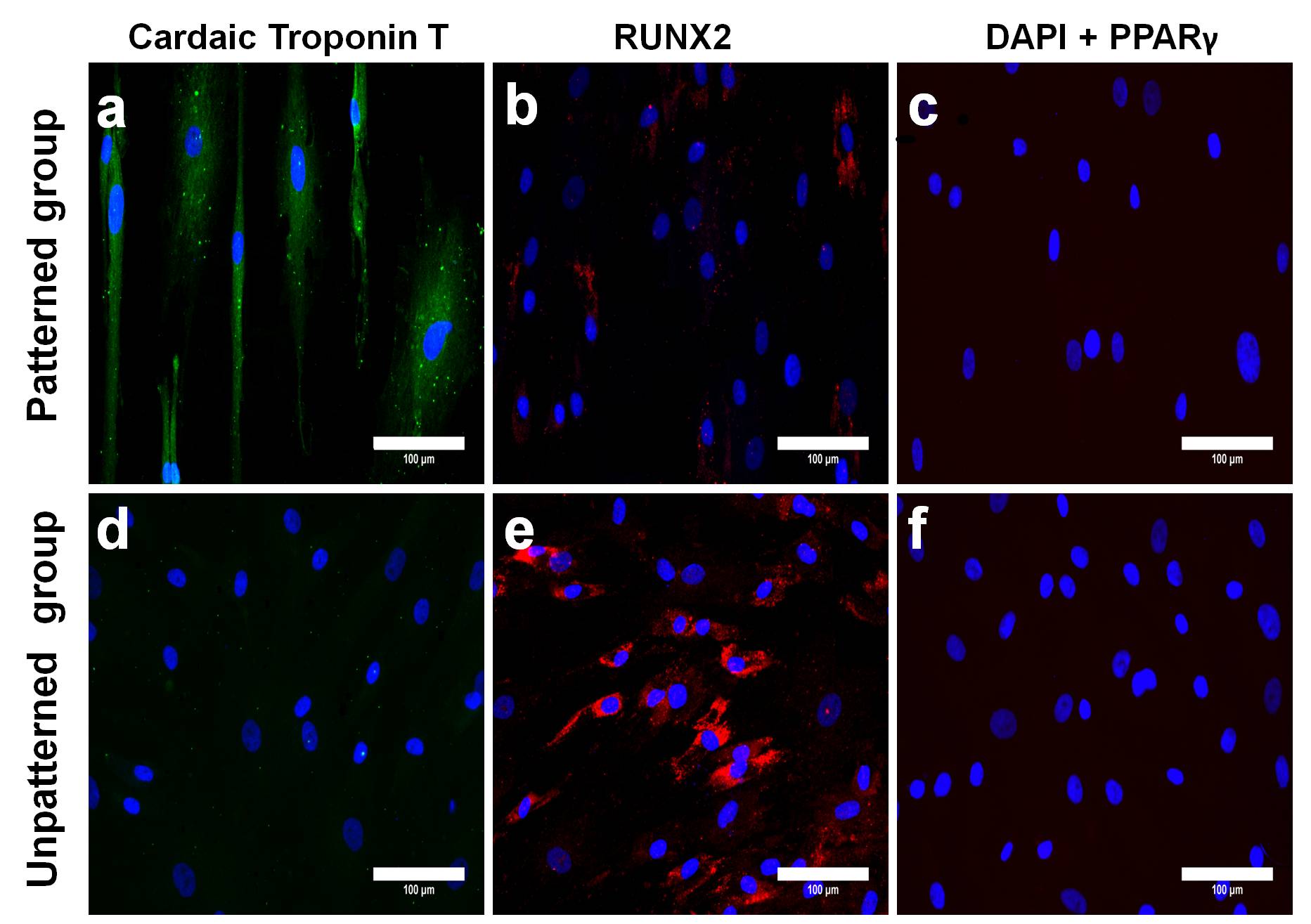
To reconfirm the adipogenic, cardiomyogenic and osteogenic lineage commitment, cell samples were immunostained to check the expression of tissue-specific markers like PPARγ (Peroxisome proliferator-activated receptors), cardiac Troponin T (cTnT) and RUNX2 respectively. We carried similar protocol as mentioned in “Immunofluorescence Staining**”** subsection to perform the immunostaining using primary antibodies of above mentioned markers. Briefly, Cell samples were incubated overnight with primary antibodies, mouse monoclonal cardiac Troponin T (1:400, Abcam), mouse monoclonal PPARγ (1:50, Santa Cruz Biotech.) and rabbit polyclonal RUNX2 (1:50, Santa Cruz Biotech) respectively at 4°C followed by labeling with the Alexa Fluor 488 goat anti mouse IgG (1:400, Molecular Probes) and Alexa Fluor 568 goat anti rabbit IgG (1:200, Molecular Probes) antibodies.

**Figure S2.** **Immunofluorescence staining of cardiac troponin T (cTnT), RUNX2 and PPARγ after 2 weeks of cell culture in normal growth medium**. Distinct expression of cTnT was observed in the patterned cells **(a)** but not in the unpatterned cells **(d)**. RUNX2 as well as PPARγ expression were absent in patterned cells **(b & C)**. Unpatterned cells showed abundant expression of RUNX2 **(e)** but no signs of PPARγ were seen **(f)**. The scale bar is 100 µm.

**
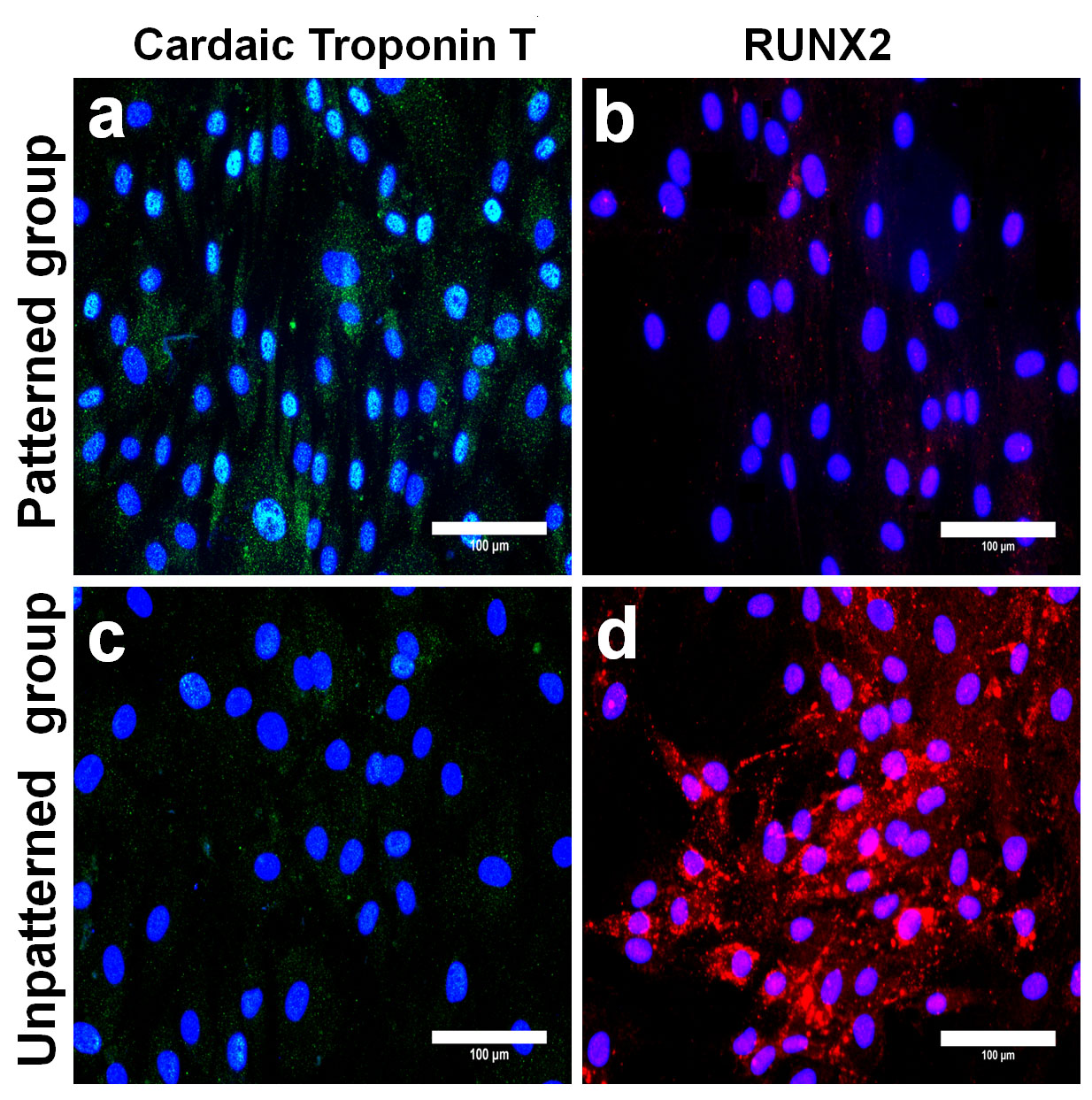
**

**Figure S3.** **Immunofluorescence staining of cardiac troponin T and RUNX2 after 21 days of hMSCs culture (14 days in normal growth medium + 7 days in osteogenic medium)**. Patterned cells stained positively for cTnT expression **(a)**, whereas unpatterned cells failed to express cTnT distinctly **(c)**. In addition, very weak expression of RUNX2 was found in patterned cells **(b)** in comparison to unpatterned cells **(d)**. The scale bar is 100 µm


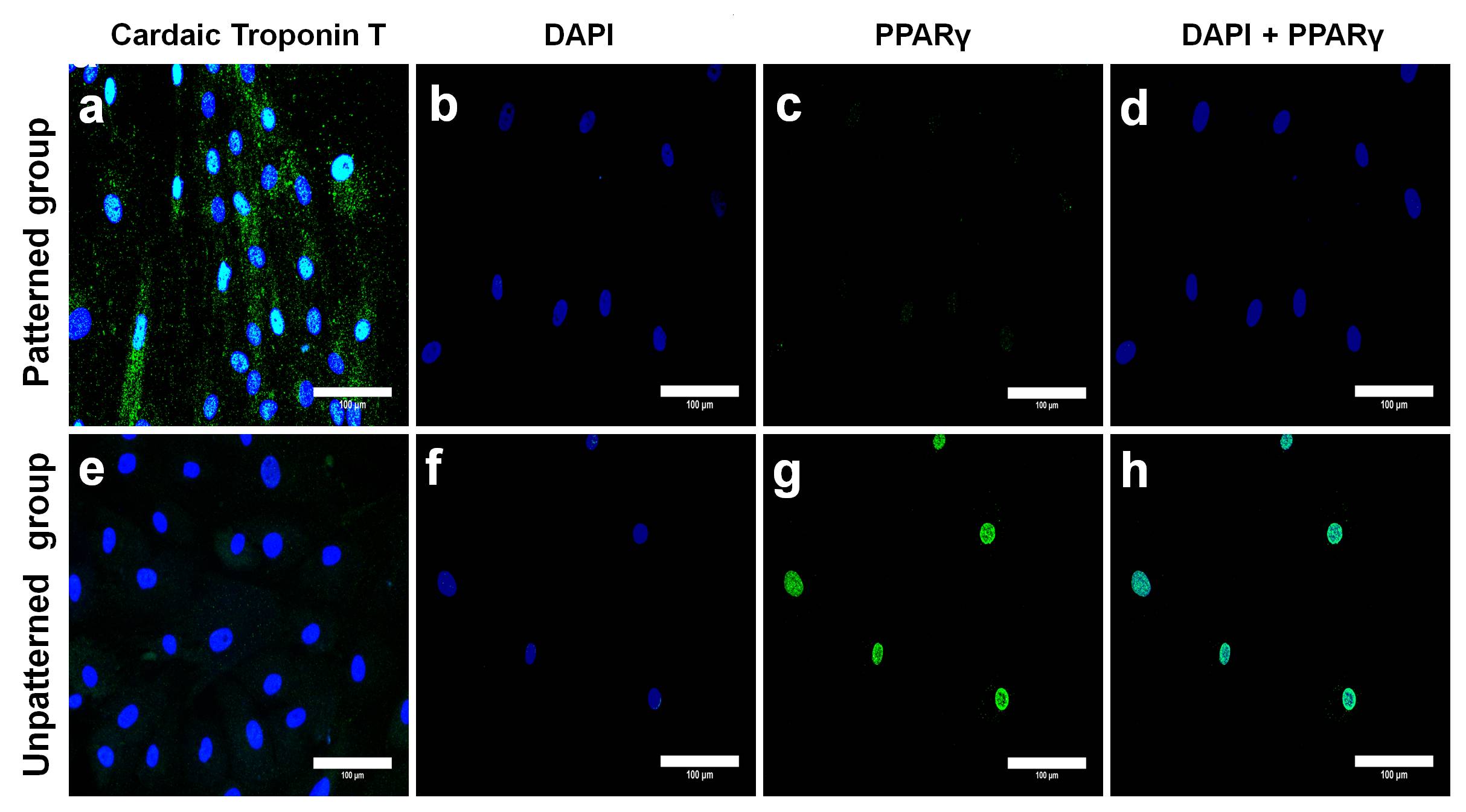


**Figure S4.** **Immunodetection of cardiac troponin T and PPARγ after 21 days of hMSCs culture (14 days in normal growth medium + 7 days in adipogenic medium)**. Up-regulation of cTnT expression was observed in patterned cells **(a)** in contrast to unpatterned cells **(e)**. PPARγ expression was found to be down-regulated in patterned cells **(c)**. On the other hand, prominent expression of PPARγ was detected in unpatterned cells **(g)**. The scale bar is 100 µm.


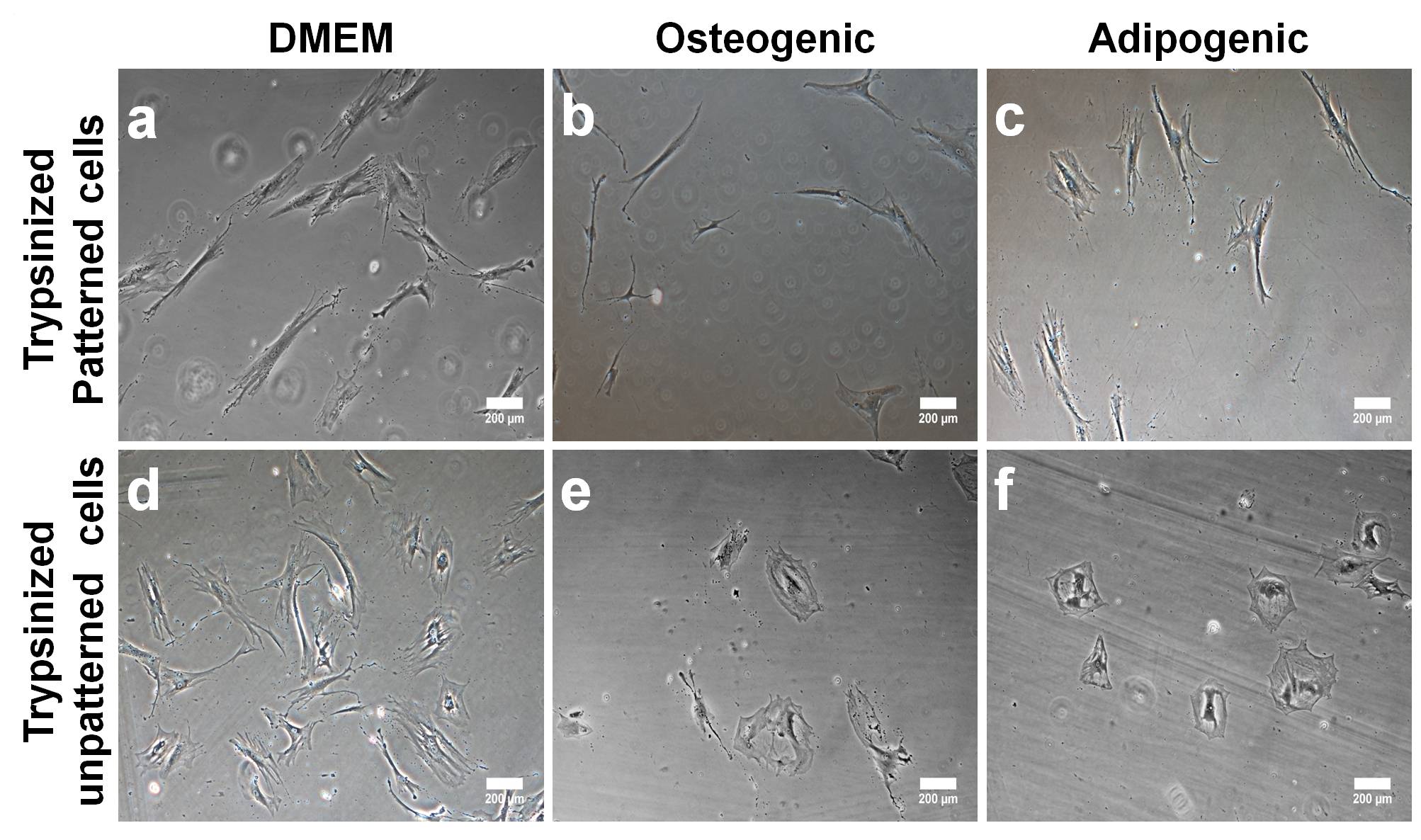


**Figure S5. Morphology of trypsinized and re-cultured hMSCs (group 3) from patterned and unpatterned groups were displayed**. Trypsinized cells were re-cultured in normal growth medium **(a, d)**, osteogenic **(b, e)** and adipogenic medium **(c, f)**. Images were captured with optical microscope after one week of re-culture. The scale bar is 200 µm.


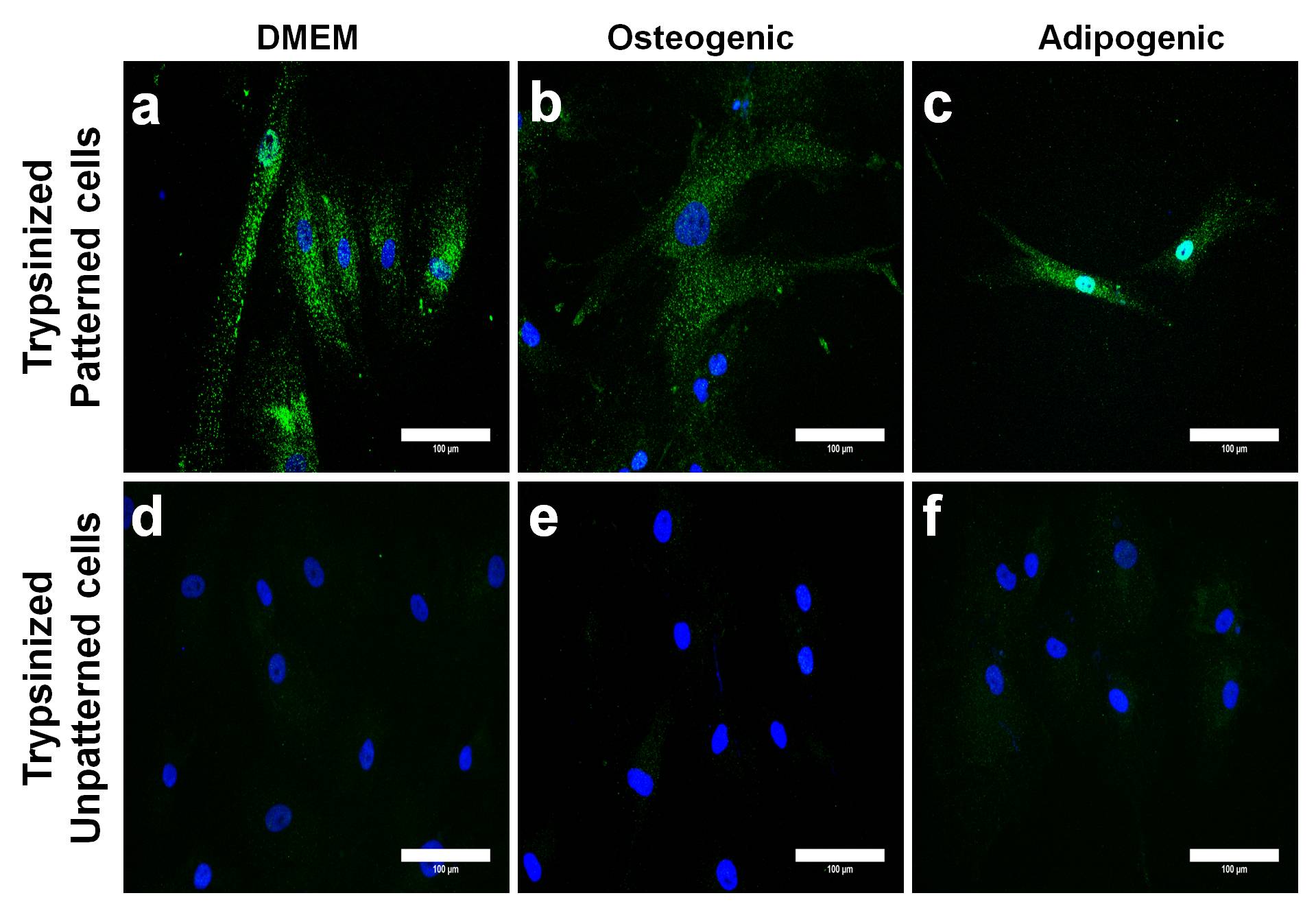


**Figure S6.** **Validation of myocardial lineage commitment of trypsinized and re-cultured hMSCs using cardiac troponin T marker (re-cultured in normal growth medium, osteogenic and adipogenic induction medium respectively)**. Trypsinized and re-cultured patterned cells maintained their myocardial lineage commitment even after 7 days of re-culture in normal growth medium **(a)**, osteogenic induction medium **(b)** and adipogenic induction medium **(c)**. Contrary to that, trypsinized and re-cultured unpatterned cells showed no signs of cTnT expression **(d, e, f)** cultured in different growth media.


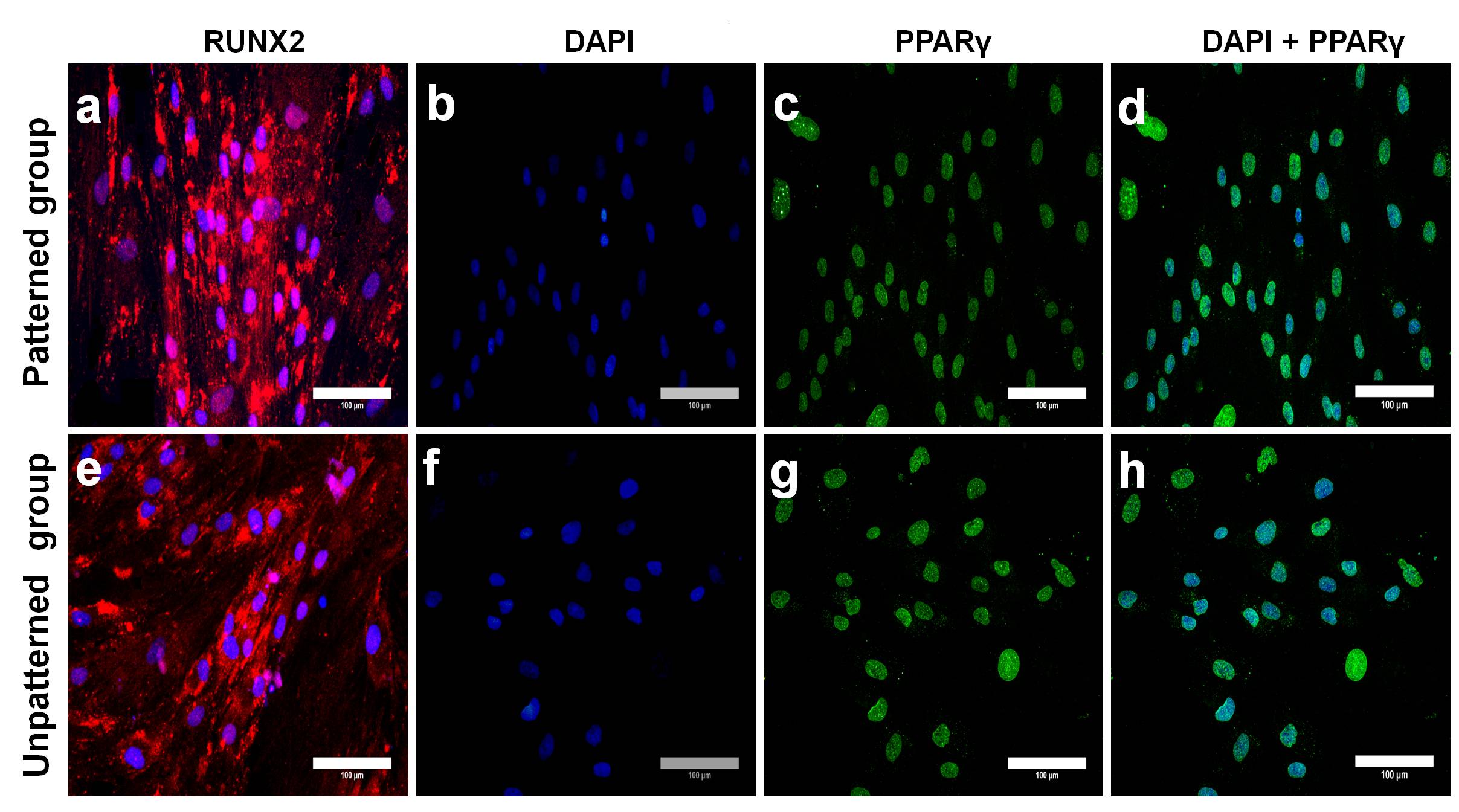


**Figure S7.** **Investigation of tissue-lineage commitment of hMSCs grown in osteogenic and adipogenic induction media for 2 weeks**. RUNX2 and PPARγ markers were used to check hMSCs commitment cultured in osteogenic **(a, e)** and adipogenic **(c, g)** media. Immunostaining results revealed that patterned and unpatterned cells showed distinct RUNX2 expression **(a, e)**. Similarly, cells from both groups cultured in adipogenic medium showed prominent PPARγ expression **(c, g).** The scale bar is 100 µm.


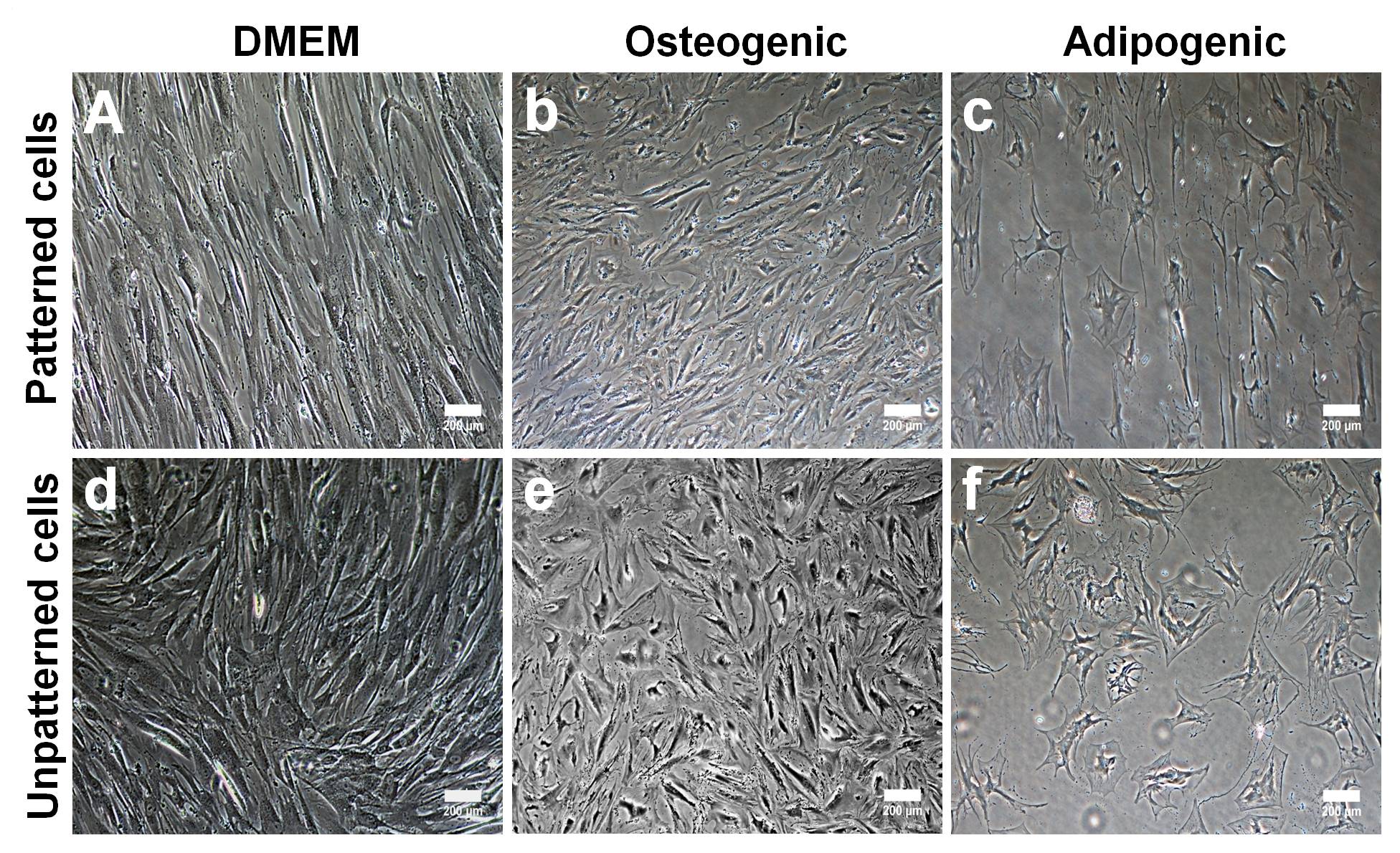


**Figure S8. Observing the morphological differences in hMSCs from both patterned and unpatterned groups**. Cells were cultured in normal growth medium **(a, d)**, osteogenic **(b, e)** and adipogenic medium **(c, f)**. Images were captured with optical microscope after two weeks of cell culture. The scale bar is 200 µm.
